# Impact of naturally leaking carbon dioxide on soil properties and ecosystems in the Qinghai-Tibet plateau

**DOI:** 10.1038/s41598-017-02500-x

**Published:** 2017-06-07

**Authors:** Xiaohong Zhao, Hongzhang Deng, Wenke Wang, Feng Han, Chunrong Li, Hui Zhang, Zhenxue Dai

**Affiliations:** 1 Key Laboratory of Subsurface Hydrology and Ecology in Arid Areas, Ministry of Education, Chang’an University, Xi’an, 710054 P. R. China; 2 Center for Hydrogeology and Environmental Geology Survey, CGS, Hebei Baoding, 071051 P. R. China; 3 Los Alamos National Laboratory, Los Alamos, New Mexico 87544 USA; 4 College of Construction Engineering, Jilin University, Changchun, 130026 P. R. China; 5 Key Laboratory of Groundwater Resources and Environment, Ministry of Education, Jilin University, Changchun, 130021 P. R. China

## Abstract

One of the major concerns for CO_2_ capture and storage (CCS) is the potential risk of CO_2_ leakage from storage reservoirs on the shallow soil property and vegetation. This study utilizes a naturally occurring CO_2_ leaking site in the Qinghai-Tibet Plateau to analog a “leaking CCS site”. Our observations from this site indicates that long-term CO_2_ invasion in the vadose zone results in variations of soil properties, such as pH fluctuation, slight drop of total organic carbon, reduction of nitrogen and phosphorus, and concentration changes of soluble ions. Simultaneously, XRD patterns of the soil suggest that crystallization of soil is enhanced and mineral contents of calcite and anorthite in soil are increased substantially. Parts of the whole ecosystem such as natural wild plants, soil dwelling animals and microorganisms in shallow soil are affected as well. Under a moderate CO_2_ concentration (less than 110000 ppm), wild plant growth and development are improved, while an intensive CO_2_ flux over 112000 ppm causes adverse effects on the plant growth, physiological and biochemical system of plants, and crop quality of wheat. Results of this study provide valuable insight for understanding the possible environmental impacts associated with potential CO_2_ leakage into shallow sediments at carbon sequestration sites.

## Introduction

Carbon Capture and Storage (CCS) is considered to be a promising technique to reduce global anthropogenic CO_2_ emissions. Natural porous sedimentary formations such as deep geological cavities, saline aquifers, depleted oil or gas reservoirs, and coal mines, are utilized to store condensed supercritical CO_2_^1–4^. However, some unexpected events like earthquake, volcanic eruption and mining cloud may release CO_2_ from the storage formations, resulting in a potential risk on groundwater, soil, vegetation, and atmosphere. It is important to asses all the potential risks and provide evidence to make sure these impacts could be tolerated. So far, many studies have been conducted to investigate the impacts of CO_2_ leakage from CCS sites on the environment and to develop monitoring techniques for detection of CO_2_ leaking^5–14^.

Since the first report relating to potential environmental impact in 2003^15^, more studies have demonstrated that leaked CO_2_ could cause some changes on underground water quality, including pH drop^16^, changes of some ions like Ca^2+^, Mg^2+^, Pb^2+^, As^2+^ and Zn^2+^ and vibration of oxidation-reduction potential of water which can induce transformation of chemical form and bioactivity of some pollutants^17–20^. Some researches have also investigated the effects of CO_2_ leakage on natural ecosystems such as the CO_2_ gas venting test site at Latera, Italy, near a volcanic area, where the soil acidification caused by long-term acid deposition was observed^5, 21, 22^. They reported a decreasing trend of soil pH, noticeable but non-significant change in mineralogy and some trace elements like As and Cr^21–23^. Moreover, Cation Exchange Capacity (CEC) change and presence of oxides like CaO, MgO, Fe_2_O_3_, and Mn_3_O_4_ were found in some studies^24–26^.

Previous studies also indicate that elevated CO_2_ can affect enzymes in soil, soil expiration, root exudates, and microbial populations either by favoring species or restricting them, depending on species and site characteristics^27–32^. In terms of vegetation ecosystem, Kruger *et al*.^33^ found that dicotyledon is more sensitive than monocotyledon for CO_2_ injection in a natural terrestrial CO_2_ vent at Leacher See, Germany. Other studies found a strong negative correlation between height of plant and CO_2_ concentration^34^ and an adverse influence of elevated CO_2_ on photosynthesis of plants, root repatriation and plant development^35–38^. Similar results were obtained in the ASGARD (Artificial Soil Gassing and Response Detection) field located at Nottingham campus, suggesting an obvious negative relationship between germination rate, growth character (plant height, leaf number, area of leave, root length and mass of shoot) and CO_2_ fluxes^39^.

The qualitative impacts found in these studies are slightly inconsistent with each other, which may be due to different soil properties and different natural or controlled vegetation conditions. The overall tendency in changes of shallow soil chemistry with CO_2_ leakage is not very clear and the effects of chronic (long-term, low level) exposure on terrestrial ecosystem is poorly characterized^5, 40^. Therefore, based on a long-term (over 10 years), naturally occurring CO_2_ leakage site at the Qinghai-Tibet Plateau, we collected detailed data of CO_2_ flux distribution, soil chemistry, response of wild plants, local crops, soil dwelling animal and microbe communities. Overall, the main objective of this study is to systematically investigate the response mechanisms of soil, plants and microbe and to evaluate the potential influence of long-term CO_2_ leakage on the whole shallow ecological environment.

## Results

A naturally occurring CO_2_ leaking site at the Qinghai-Tibet plateau was investigated to explore the effects of elevated CO_2_ on the environment. Due to complicated reactions of the CO_2_-water-soil system (including dissolution, exchange, hydration, hydrolysis and corrosion, etc.), CO_2_ invasion results in changes of soil properties, like pH fluctuation, reduction of nutrients such as nitrogen, phosphorus, and some changes in soluble ions. Transformation of soil minerals was confirmed by the XRD test, demonstrating that CO_2_ incursion could enhance the crystallization of soil in which CaCO_3_ and anorthite increase is more pronounced.

In terms of vegetation, a large CO_2_ injection caused adverse effects on the plant community distribution, plant growth, physiological and biochemical systems of plants, and crop quality of wheat. The possible reasons are pH change, lack of nutrients like available N, P, inhibition of soil respiration induced by replacement of O_2_ with excess CO_2_, and depression of photosynthesis in plant leaves. However, moderate CO_2_ injection could improve the plant growth and development and enhance the fat content in rapeseeds and starch content in potatoes.

Under CO_2_ stress, both volumes and structures of soil dwelling animals varied, some new microbes appeared, and some others vanished. Compared with bacteria, the amount of fungus was quite stable where the variation of community structures was distinguishable.

### Carbon dioxide profile

Table 1 showed the CO_2_ concentrations and temperatures of soil in different points along with 5 transects, and soil sampling strategy. Overall, most CO_2_ concentrations in soil close to the vents and eruptible spring were pretty high, decreasing with the distance to the vents and eruptible spring, and the local background value of CO_2_ ranges 400–700 ppm. In addition, there is a significant positive relationship between CO_2_ concentrations and soil temperatures. Base on the data, most CO_2_ concentrations in soil were less than 5000 ppm. Through linear-regression analysis, there was a approximately linear relationship between CO_2_ concentrations and soil temperatures, with a sharp slope. In the range of 5000–60000 ppm, a roughly linear correlation existed with a small gradient.

**Table 1:** CO_2_ profiles of the field.

Profile	No.	Coordinates		CO_2_ (ppm)	T (°C)		Profile	No.	Coordinates		CO_2_ (ppm)	T (°C)	
		X (m)	Y (m)						X (m)	Y (m)			
1	01	3	5	2170	5.0		2	20	0	50	3170	6.7	
	02	3	10	42000	8.0			21	5	50	52000	8.2	
	03	3	15	6000	6.5	√		22	10	50	1410	5.9	
	04	3	20	3280	5.7			23	15	50	1710	5.2	
	05	3	26	3010	4.8			24	20	50	440	5.0	
	06	3	30	18000	5.9	√	3	25	10	75	650	6.7	
	07	3	35	1930	4.8			26	15	75	510	5.1	
	08	3	40	1850	4.7			27	20	75	490	3.2	
	09	3	45	1460	5.8		4	28	0	15	42000	6.9	
	10	3	50	23000	7.1	√		29	5	15	>112000	7.5	√
	11	3	55	15000	8.1			30	10	15	3130	7.9	
	12	3	65	12000	5.7	√		31	15	15	4100	7.5	
	13	3	70	1260	5.4		5	32	15	20	2810	7.9	
	14	3	75	2100	5.8			33	15	25	17000	7.5	
	15	3	80	1340	5.7			34	15	30	2040	7.0	
	16	3	85	870	7.0		Blank	001	−20	77	680		
	17	3	90	530	6.4			002	Around 300 m north to the research field		500		√
	18	3	95	690	7.0								
	19	3	100	695	5.1								

Note: the test depth of CO_2_ concentration and temperature is 20–30 cm; ‘√’ means taking soil sample in this point.

### Soil physical and chemical properties

Soil properties are very important for plants and microorganisms in soil. Based on the CO_2_ concentrations at sampling points, six typical soil samples marked as S002 (Blank), S03, S06, S10, S12 and S29 were selected to investigate the variation of soil properties under CO_2_ invasion with different concentrations of 500 ppm, 6000 ppm, 18000 ppm, 23000 ppm, 12000 ppm, and >112000 ppm respectively.

**Figure 1:**
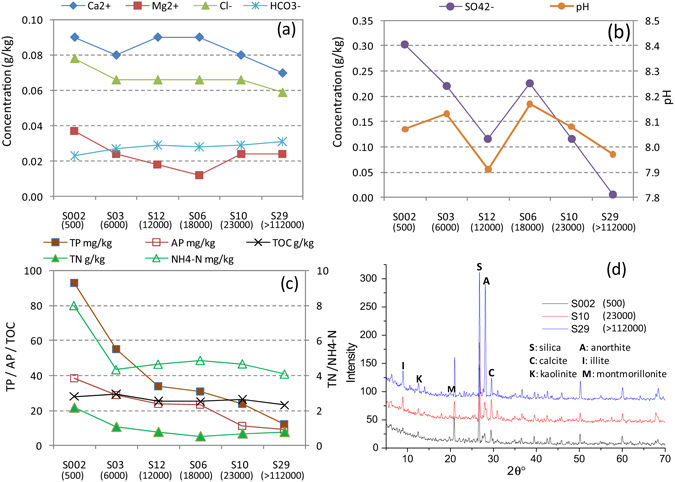
Soil physical and chemical properties (the numbers in bracket are CO_2_ concentrations; unit: ppm).

Figure 1 illustrates the results of soil chemistry. First of all, pH is a vital parameter indicating the quality and maturity of soil. In general, pH lowers than 4 or higher than 9 will restrain or destroy the metabolism of plants. Moreover, pH can affect the adsorption of ions like Ca, Mg, N, P and K by plants. In this case, pH range was 7.91–8.17 (Fig. 1(b)), no apparent change under CO_2_ incursion, but having a slight decrease in S12 and S29 compared with blank soil.

The variation of main exchangeable ions in soil such as Ca^2+^, Mg^2+^, Cl^−^ and HCO_3_^−^ under CO_2_ exposure was shown in Fig. 1(a). Ca^2+^ concentrations were quite stable in soil with the range of 71–92 mg/kg, but the minimum was observed in S29. In contrast, soluble Mg^2+^ has lower concentration of 12–37 mg/kg, with a decreasing trend under CO_2_ invading, but the minimum showed in S06. Negative ion Cl^−^ has an abundance of 59–78 mg/kg and a slight reduction was observed with elevated CO_2_. In the Fig. 1(b), the concentration of SO_4_^2−^ in blank soil was as high as 302 mg/kg, but showed a sharp drop under CO_2_ injection as a whole. However, there was an extraordinary increasing from S12 to S06. These concentration variations of SO_4_^2−^ are possibly due to the soil heterogeneity and/or measurement uncertainty. Further study is needed for evaluating the impact of the soil heterogeneity and measurement errors on the observed concentration variations. CO_3_^2−^ was not detected, suggesting alkalization of soil was not high. The concentration of HCO_3_^−^ ranged 23–31 mg/kg, with a mild enhancement possibly induced by CO_2_.

As shown in Fig. 1(c), total phosphorus (TP) in soil was between 12–93 mg/kg, with a significant reduction trend along with increasing CO_2_ concentration. Similarly, available phosphorus (AP) ranging from 9.2–38.2 mg/kg showed a decreasing trend under CO_2_ injection. The content of total nitrogen (TN) in soil was 0.53–2.17 g/kg and an obvious decrease was observed with increasing CO_2_ concentration. NH_4_-N amount in soil was quite small varying just 4.09–7.99 mg/kg. Compared with blank soil, NH_4_-N content dropped to near half when CO_2_ concentration was 6000 ppm in S03, but no big difference between other samples. The amount of total organic carbon (TOC) was 23.13–29.28 g/kg, no apparent change linked with CO_2_ injection.

Figure 1(d) is the image of the XRD, indicating the mineral components in the soil. Based on XRD standard diffraction patterns, the peaks locating in 27°, 28° and 29.5° were identified as the characteristic peaks of silica-SiO_2_, anorthite-(Ca,Na) (Al,Si)_2_Si_2_O_8_ and calcite-CaCO_3_, respectively. The main characteristic peaks of Illite-(K,H_3_O)Al_2_Si_3_AlO_10_(OH)_2_ are located at 2θ angle of 8.8°and 27°, having some overlap with SiO_2_. 2θ of 12.5° is the strongest peak of kaolinite- Al_2_SiO_5_(OH)_4_. The little peak in 19.7° is the character peak of montmorillonite-Ca_0.2_(Al,Mg)_2_Si_4_O_10_(OH)_2_·4H_2_O. Chlorite cannot be found in this test. Through comparing the location and intensity of the peaks, it can be seen that the main component of soil is SiO_2_ and under CO_2_ exposure, the crystallization of the soil was improved. Simultaneously, the peak intensities of illite and kaolinite were enhanced as well, but montmorillonite nearly disappeared.

### Plant community distribution and apparent character

CO_2_ concentrations in central soil of four quadrats named as P1, P2, P3 and P4 were 1100 ppm, 64900 ppm, >100000 ppm and 6000 ppm, respectively, under a test depth of 20–30 cm.

**Figure 2:**
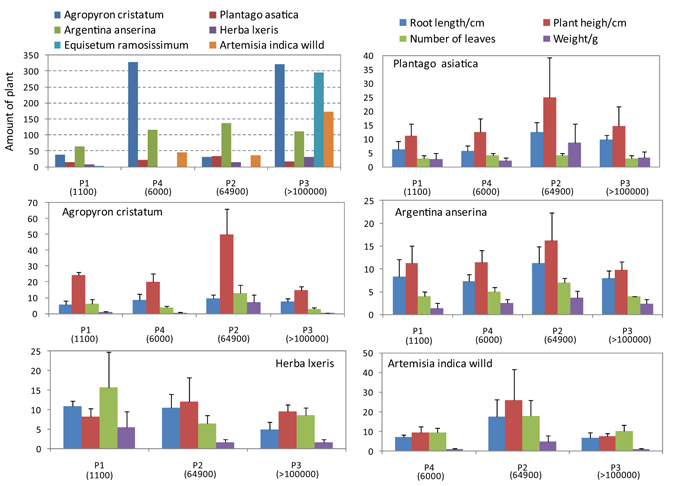
Plant community distribution (**a**) and apparent characters of Agropyron cristatum, Plantago asiatica, Argentina anserina, Artemisia indica willd and Herba lxeris (**b**–**f**).

As shown in Fig. 2(a), totally, there was a trend of enhancement on total vegetation coverages and quantities of plants along with increasing CO_2_ concentration. Among them, the amounts of *Agropyron cristatum, Equisetum ramosissimum* and *Artemisia indica Willd* increased obviously. There was a slight increase on number of *Herba lxeris*, whereas stable on *Agropyron cristatum* and *Plantago asiatica*. By analyzing CO_2_ concentrations, it should be noted that plant amounts enhanced with increasing CO_2_ concentration, especially significant in P3 plot where *Equisetum ramosissimum*, *Artemisia indica Willd* and *Herba lxeris* were more abundant than other plots. But there was no obvious relationship between CO_2_ concentration and plant community distribution.

Figure 2(b–f) also illustrates the results of apparent plant character parameters such as total weight, stem length, number of leave and root length. It can be concluded that except *Herba lxeris* and *Equisetum ramosissimum*, middle CO_2_ concentration of 66400 ppm in P2 could benefit other plant growth. However, elevated CO_2_ over 100000 ppm could bring inhibition. For *Herba lxeris*, except plant height, invaded CO_2_ always causes adverse effects on root length, number of leaves and weight.

### Physiological and biochemical properties of *Argentina anserina* and *Plantago asiatica*

*Argentina anserina* and *Plantago asiatica* were chosen to investigate the influence of elevated CO_2_ on physiological system and results were listed in Table 2.

**Table 2:** Physiological and biochemical characteristics of plants under CO_2_ incursion.

Plant	Area	CAT (U/mg)	POD (U/mg)	SOD (U/mg)	PRO (ug/mg)	Chl a (mg/g)	Chl b (m/g)	Caro. (mg/g)	Sugar (umol/g)	Protein (mg/g)
*Argentina anserina*	P1 (1100)	413.01 ± 261.84	75.86 ± 49.23	450.94 ± 83.20	34.29 ± 2.80	0.528 ± 0.126	0.201 ± 0.037	0.143 ± 0.029	0.567 ± 0.036	4.732 ± 1.198
	P4 (6000)	747.98 ± 154.03	33.34 ± 10.93	258.91 ± 86.32	44.85 ± 9.77	0.161 ± 0.051	0.063 ± 0.023	0.069 ± 0.026	0.224 ± 0.019	5.918 ± 0.080
	P2 (64900)	246.40 ± 89.09	53.86 ± 19.43	140.72 ± 35.94	17.60 ± 7.04	0.412 ± 0.073	0.165 ± 0.023	0.130 ± 0.020	0.230 ± 0.009	6.553 ± 0.046
	P3 (>100000)	609.08 ± 297.68	49.91 ± 20.01	247.74 ± 34.08	56.44 ± 31.53	0.177 ± 0.100	0.073 ± 0.038	0.088 ± 0.009	0.221 ± 0.007	6.616 ± 0.106
*Plantago asiatica*	P1 (1100)	103.69 ± 5.69	95.81 ± 68.08	364.94 ± 47.51	52.08 ± 28.16	0.109 ± 0.034	0.042 ± 0.015	0.037 ± 0.013	0.213 ± 0.011	7.465 ± 1.228
	P4 (6000)	53.53 ± 18.75	129.39 ± 52.12	256.65 ± 12.67	50.66 ± 10.84	0.126 ± 0.064	0.049 ± 0.029	0.035 ± 0.009	0.267 ± 0.012	2.238 ± 0.290
	P2 (64900)	59.74 ± 23.52	140.05 ± 40.05	244.47 ± 84.21	6.02 ± 2.72	0.076 ± 0.005	0.030 ± 0.001	0.031 ± 0.007	0.225 ± 0.018	2.363 ± 0.611
	P3 (>100000)	67.49 ± 13.38	76.63 ± 2.13	261.43 ± 33.09	45.28 ± 32.75	0.120 ± 0.026	0.046 ± 0.013	0.025 ± 0.019	0.291 ± 0.008	2.830 ± 0.620

Note: CAT: catalase; POD: peroxidase; SOD: superoxide; PRO: proline; Chl: chlorophyll; Caro: carotenoids; the number in the brackets under P1 to P3 is the measured CO_2_ concentration (unit: ppm).

The immune system of plant consists of SOD, CAT and POD, providing crucial abilities to clear superoxide radicals, H_2_O_2_ and peroxides to inhibit and reduce the formation of hydroxyl radicals^41^. Normally, the concentration of radicals in plant is quite low, but will increase under some environmental stress, bringing adverse effects on plant growth like promoting plant senescence^42^. Simultaneously, through enhancing the activity of antioxidase, the injury of organism can be reduced. PRO is a part of protein and its content will enhance when suffering stress as well. Therefore, measuring the activities of SOD, CAT, POD and PRO can identify if a plant suffers stress. By analyzing the results of SOD, CAT, POD and PRO, *Argentina anserina* in point P2 with CO_2_ concentration of 64900 ppm showed the lowest level of stress. Although CO_2_ concentration in P1 was only 1100 ppm, *Argentina anserina* still showed high stress resistance which possibly resulted from the acidification of surface soil caused by corrosion of acid eruptible spring water. Similar results were observed on *Plantago asatica* with high SOD, CAT, POD and PRO concentration in P1 plot. Compared with *Argentina anserina*, *Plantago asiatica* showed a better tolerance on CO_2_ injection.

In order to explore the effect of CO_2_ exposure on photosynthesis a crucial process for plant growth, photosynthetic pigments including chlorophyll a, chlorophyll b and carotenoid were monitored. It can be seen that there are no clear trends along with increasing CO_2_ concentration, but photosynthesis of *Argentina anserina* in P1 was stronger than other plots, indicating heavy stress induced by soil acidification. No big differences were obtained in *Plantago asiatica*, suggesting good tolerance of it.

The changes of sugar and protein concentration indicate the metabolic function of plants. Along with elevated CO_2_, sugar concentrations in *Argentina anserina* decreased sharply in P4 but quiet stable from P4 to P3. Simultaneously, protein levels consistently increased with increasing CO_2_, although they were similar in P2 and P3. However, adverse trends were observed in *Plantago asiatica*. In total, because of acidification in plot P1, plants showed the highest stress resistance. Except P1, a slight increasing trend of diverse indexes still could be observed along with elevated CO_2_, meaning an injury caused by CO_2_. Comparing two plants, *Argentina anserina* is more sensitive for stress than *Plantago asiatica*.

### Crop quality

**Figure 3:**
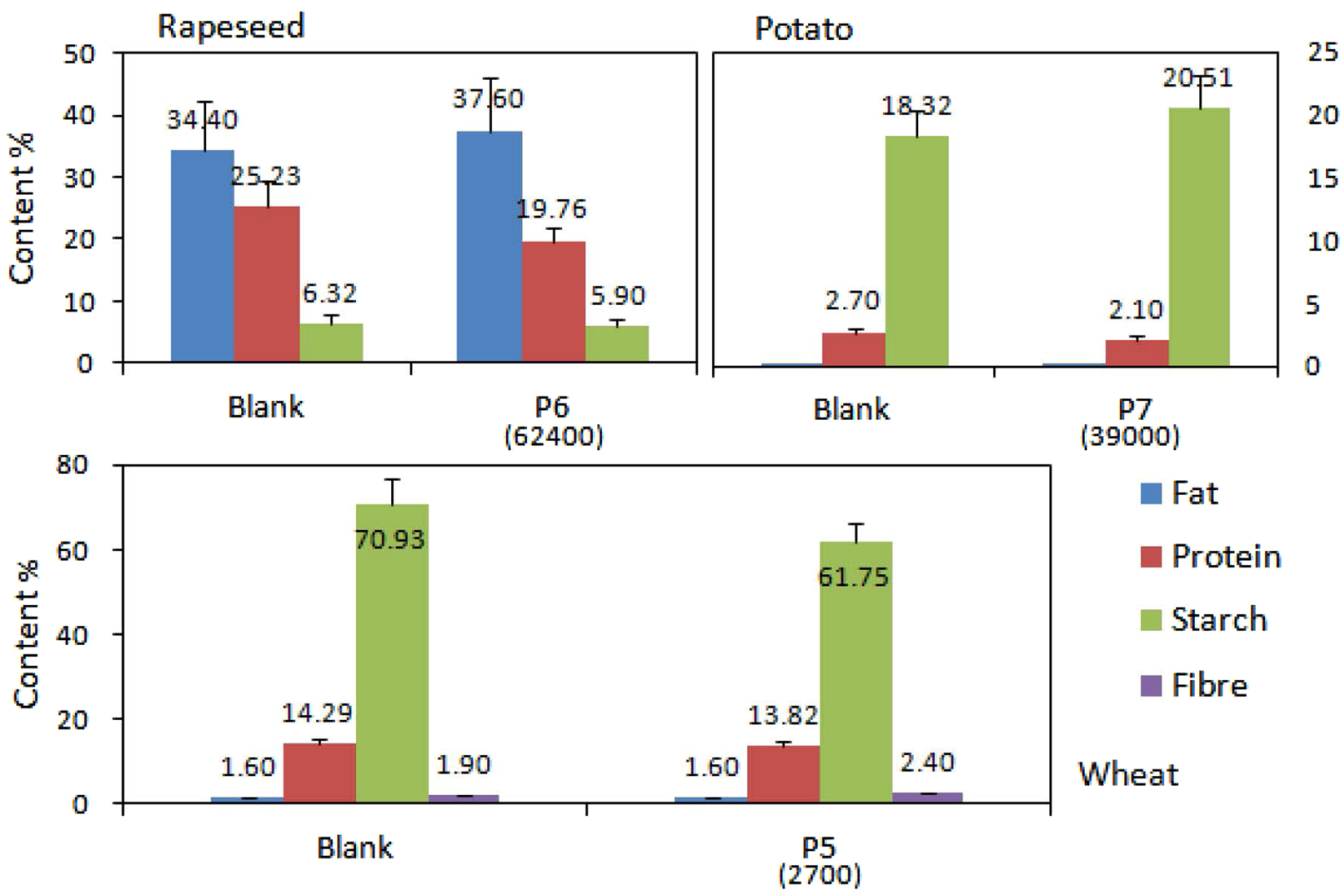
The effect of elevated CO_2_ on quality of crop fruits.

Surrounding the CO_2_ field, some typical local crops were picked to analyze the effect of CO_2_ on crop quality. As shown in Fig. 3(c), ear of wheat, rapeseed and potato were taken in point P5, P6 and P7 with CO_2_ concentrations of 2700 ppm, 62400 ppm and 39000 ppm, respectively. Blank samples were obtained from local farmland which is in the same valley with the CO_2_ research field, less than 1 km away from the CO_2_ field.

From Fig. 3, under elevated CO_2_, fat content in rapeseed enhanced from 34.4% to 37.60%, whereas protein and starch contents decreased obviously. Starch concentration in potato increased around 2.2%, but protein decreased slightly. In contrast, fat, protein and dietary fibre in ear of wheat were quite stable, but starch content decreased from 70.93% to 61.75%, indicating elevated CO_2_ could bring adverse effects on wheat quality.

### Soil dwelling animal

Through soil dwelling animal investigation consisting of nine blank points and nine CO_2_ invaded points, it was found that in the blank points, soil dwelling animals mainly included earthworm, ant, “white worm”, pillbug, millipede and “yellow worm”, whereas only ant, pillbug and “yellow worm” appeared in CO_2_ invaded points with a concentration range of 6000–110000 ppm.

Under high CO_2_ concentrations over 110000 ppm, soil dwelling animals totally disappeared, revealing a significant adverse effect of CO_2_ on animal diversity which indicates deterioration of soil condition. Comparatively, earthworm and millipede are more sensitive for CO_2_ incursion.

### Microbiology

**Figure 4:**
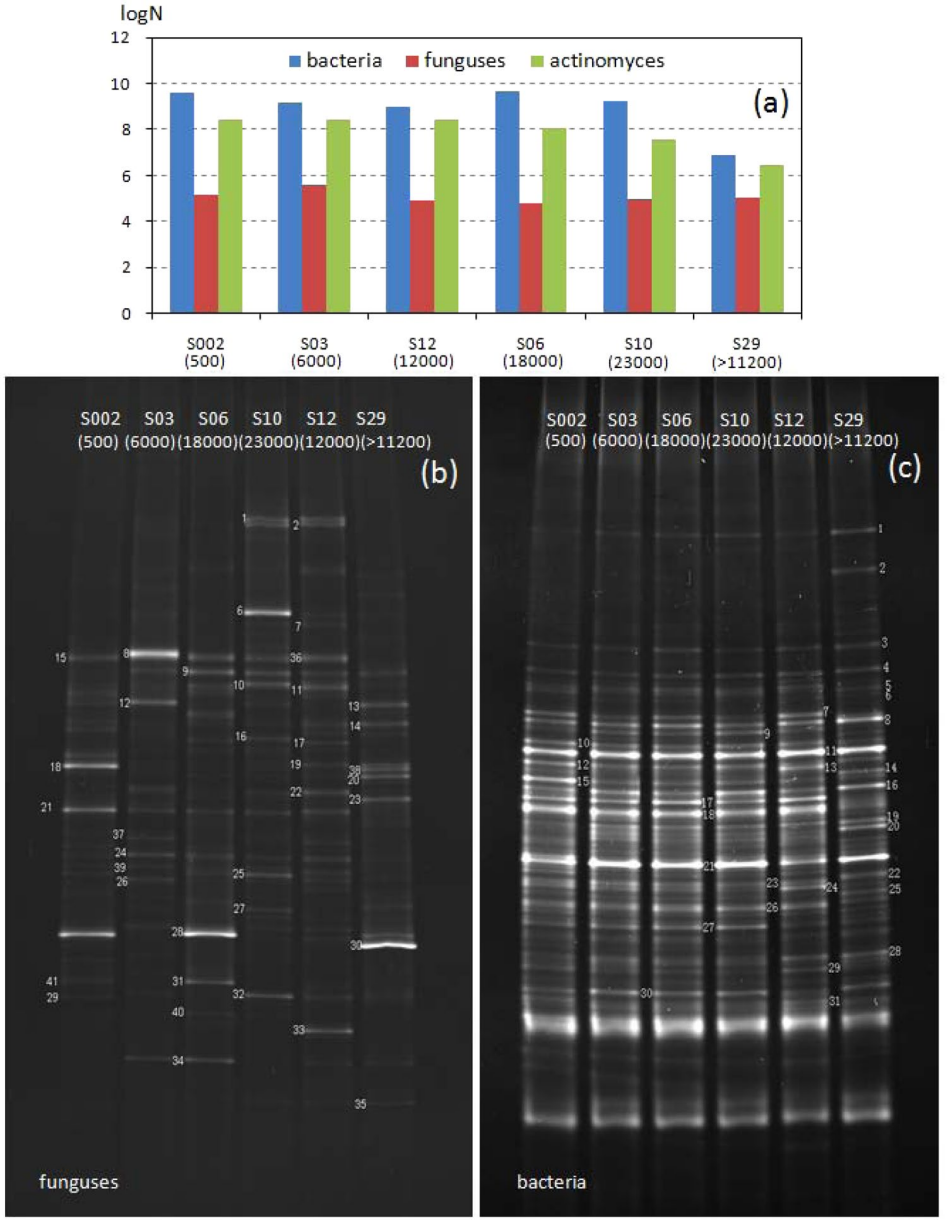
Amounts of bacteria, funguses and actinomyces under CO_2_ invasion (**a**), and 16 S rDNA-DGGE fingerprint patterns of the soil funguses and bacteria (**b**,**c**).

By using plate count method, the amounts of bacteria, funguses and actinomyces were investigated and the results are shown in Fig. 4(a).

Obviously, the tolerance of different microbes was not the same. In total, funguses showed the best tolerance since the amounts in different soil were quite similar. Bacteria and actinomyces are more sensitive to CO_2_ invasion, and there was two orders of magnitude difference between blank and high CO_2_ concentration point.

In order to explore the variation of microbial community, DGGE test was conducted and the obtained fingerprint patterns are shown in Fig. 4(b,c). It can be seen that fungus communities varied obviously under different CO_2_ concentrations. Compared with blank soil (S002, 500 ppm), new dominante bands of 8 and 12 emerged in soil S03 (6000 ppm). When CO_2_ concentration increased, new dominate bands of 1 and 2 appeared in S10 (23000 ppm) and S12 (12000 ppm), and band 6 only observed in S10, which means some new kind of fungus presented in the soil. Band 18 was obtained only in the blank soil, and nearly vanished under CO_2_ injection. Lighting level of band 21 decreased in CO_2_ invaded soil, indicating the amount of this kind of fungus decreased significantly under CO_2_ invasion. In total, it can be concluded that elevated CO_2_ could result in mutation of the fungus gene, producing some new funguses which can only exist in a certain CO_2_ concentration range.

In contrast to fungus, bacterial communities were quite stable, only two new dominate bands of 2 and 14 appeared in S29 (>112000 ppm), suggesting a higher adaptability and stability of bacterial communities as compared to fungus. Along with CO_2_ concentration increasing, the amount of bacteria represented with band 1, 19, 20 and 25 enhanced, whereas the amount of bacteria represented with band 10, 15 and 26 decreased. When CO_2_ elevated over 112000 ppm, band 7, 9, 18, 27, 29 and 31 disappeared, indicating CO_2_ invasion not only can suppress the growth but also can result in extinction of some bacteria.

In summary, soil, plants and microbes build up a complex and inner connected ecosystem in which they affect each other. In a certain range, excess CO_2_ could result in a change of soil conditions, leading to adverse effects on plant growth and microbe community distribution.

## Discussion

Soil is the foundation for plants and microorganisms and it is a very complicated system consisting of three phases of gas, liquid and solid. Normally, the volumetric proportions of air, water and minerals are around 20–30%, 20–30% and 45%, respectively, and 5% left are organics^43^. The solid phase of soil consists of two main parts which are cements such as salts, hydroxides, oxides, and organics etc., and minerals including primary minerals like quartz, feldspars, mica and secondary minerals, especially secondary silicates such as illite, kaolinite and montmorillonite. Illite has a high cation exchange capacity and a high content of potassium. If soil is enriched with montmorillonite, plant growth will be inhibited by lack of available water. Comparably, kaolinite can provide more water but less nutrition^43^. Therefore, as a complex multiphase system, soil physical and chemical properties are influenced by many factors such as water content, gas composition and minerals.

When CO_2_ invades soil, H_2_CO_3_ is produced through CO_2_ dissolution in water and pH of soil drops because H_2_CO_3_ splits into H^+^ and HCO_3_^− ^^44^. In general, a high concentration of CO_2_ introduced into soil will inevitably induce the change of CO_2_ content in soil gas phase and cause the enhancement of H^+^, HCO_3_^−^ and CO_3_^2−^ which breaks precipitation-dissolution equilibrium, leading to variations of mineral type and content, porosity and permeability.

As described above, the pH of six soil samples under CO_2_ exposure with different concentrations ranged from 7.91–8.17, which was mainly affected by soluble minerals in soil. According to the results obtained in this study, after a CO_2_ injection, pH varied slightly, no obvious relationship with CO_2_ concentration. The possible reason is that pH of air-dried soil resulted from the concentration of ions extracted by water, which showed a small change in the test.

Because soil is a complicated gas-water-solid system, through dissolution, exchange, hydration, hydrolysis and corrosion processes, mineral type and concentration can be changed and some ions like Ca^2+^, Mg^2+^ vary as well^45–47^. In this study, under CO_2_ incursion, slightly decreasing trends can be observed in Ca^2+^, Mg^2+^, and Cl^−^ concentration changes. Hypothetical reason could be more production of insoluble compounds such as calcite and sulfates, resulting in the decrease of soluble ions. Concentration of HCO_3_^−^ was quite stable, no significant change. Nutrient components like TN, NH_4_-N, TP and AP all declined with different magnitudes, suggesting CO_2_ injection could induce the reduction of nutrients. The possible reasons cannot be identified since the variation of nutrients is quite complicated, not only relating to the soil characteristics but also connecting with activities of living beings like plant and microbe etc. XRD test showed the small changes of mineral crystallization and mineral types, providing a reference for assessment of the effect of CO_2_ injection on soil minerals. Due to the uncertainty in the XRD measurements and the soil heterogeneity, further next-step investigation is required for better understanding the changes of the mineral crystallization.

Plant growth is related to many factors such as soil property, weather conditions (precipitation, evaporation, temperature and solar radiation etc.), landform, vegetation and land-use type. Due to more than 10 years of natural CO_2_ leakage, there were no crops planted by humans in this research field, only some wild grasses and plants emerged in the east part of Area 1. As described previously, there was a big difference between the four selected points of plant investigation. Since a serious acidification induced by the flooding of acid spring water was observed in point P1, there was the smallest amount of plants compared with the other three points, and *Artemisia indica willd* can not be found in this point. The character of different plants like root length, plant height, number of leaves and weight showed the different responses under CO_2_ exposure. Generally, moderate CO_2_ intrusion can enhance the plant growth and development, but under high concentration (>100000 ppm), CO_2_ would lead to obviously adverse effects. Our findings are consistent with other literature which reported elevated soil CO_2_ adversely affects plant height, leaf number^39^, root growth and function^36, 48^ and shoot biomass^49^. They suggested that the possible reason is the substantial depletion of soil O_2_ induced by CO_2_ injection which could result in hypoxic conditions, inhibiting respiration of roots. Additionally, from the soil chemistry trail, it should be noted that elevated CO_2_ could cause a reduction of nutrients and pH change which in turn could result in variations of soluble salts and affect the plant growth.

Photosynthesis is a vital process for plant growth, which transforms CO_2_ and H_2_O into carbohydrates using solar energy by chloroplastid. In general, plant senescence and deficiency of mineral elements for photosynthetic pigment production will cause the reduction of pigment concentration, resulting in attenuation of photosynthesis which affects plant growth^50^. From our results, CO_2_ injection can improve the photosynthesis of *Argentina anserina* and *Plantago asiatica* under concentration of 64900 ppm and 6000 ppm, respectively, but showed the inhibition under higher concentration of >100000 ppm. Protein and surge are important for metabolism in plants. It is interesting that along with increasing CO_2_ content, protein in *Argentina anserina* increased but surge decreased, and the adverse trends were observed in *Plantago asiatica*, suggesting the different responses of different plants.

When plants suffered stress such as drought and excess salt and alkali, the protective enzyme system consist of SOD, CAT, POD and PRO will increase to eliminate injury^51^. However, no distinct variation tendency of SOD, CAT and POD concentrations linked with CO_2_ were observed in this study, possibly resulting from different resistance thresholds of SOD, CAT and POD. Therefore, under stress, different plants have different reactions to reduce injury, such as CAT and PRO increasing in *Argentina anserina*, and POD and SOD enhancement in *Plantago asiatica*.

Elevated CO_2_ could bring change to soil conditions and plant root systems^52, 53^, directly leading to the variation of soil dwelling animals and microorganisms. Normally, if there is a change of environmental condition or invasion of foreign compounds, soil microbes will try to adapt to the new environment and eliminate the injury through physiological regulation and genetic variation^52, 54, 55^. Temperature, moisture and especially pH are crucial for a microorganism community, in which variations of pH could result in enrichment of certain bacteria and reduction of others^30^. Also, changes of oxygen content and the supply of nutrients for microbial growth may be another reason for soil microbial community structure change. These findings are confirmed by our results as well.

Although this study was conducted in a natural CO_2_ leaking site with uncontrolled and flexible CO_2_ levels in the soil, it can still be concluded that elevated CO_2_ resulted in a change of soil properties like pH, soluble salts, minerals and nutrients, inducing changes of plant growth and development, and changes of microbial community structure. Undoubtedly, the whole ecosystem is complicated and interconnected, and the soil, plants and microbes could affect each other. Therefore, this study only revealed the exterior phenomena between elevated CO_2_ and the ecosystem. But as an analog of CCS site, the results from this study are significant for assessing the environmental risk of CCS leaking as well as inspiring for developing indicator of CO_2_ leaking. Absolutely, further investigation on the detailed mechanism of stress resistance of plants and microbes, and long term trials for evaluation of environment change induced by CO_2_ incursion is necessary and required in future work.

## Methods

### Study site

This study was conducted at the CO_2_ research field, in Ping’an County, Qinghai Province, China (36.48°N, 102.40°E), at an altitude of 2100 m, which belongs to the Qinghai-Tibet Plateau. This area is a typical continental semi-arid zone characterized by a mean annual precipitation of 248–600 mm and a mean annual temperature of 0.3–6.4 °C.

**Figure 5:**
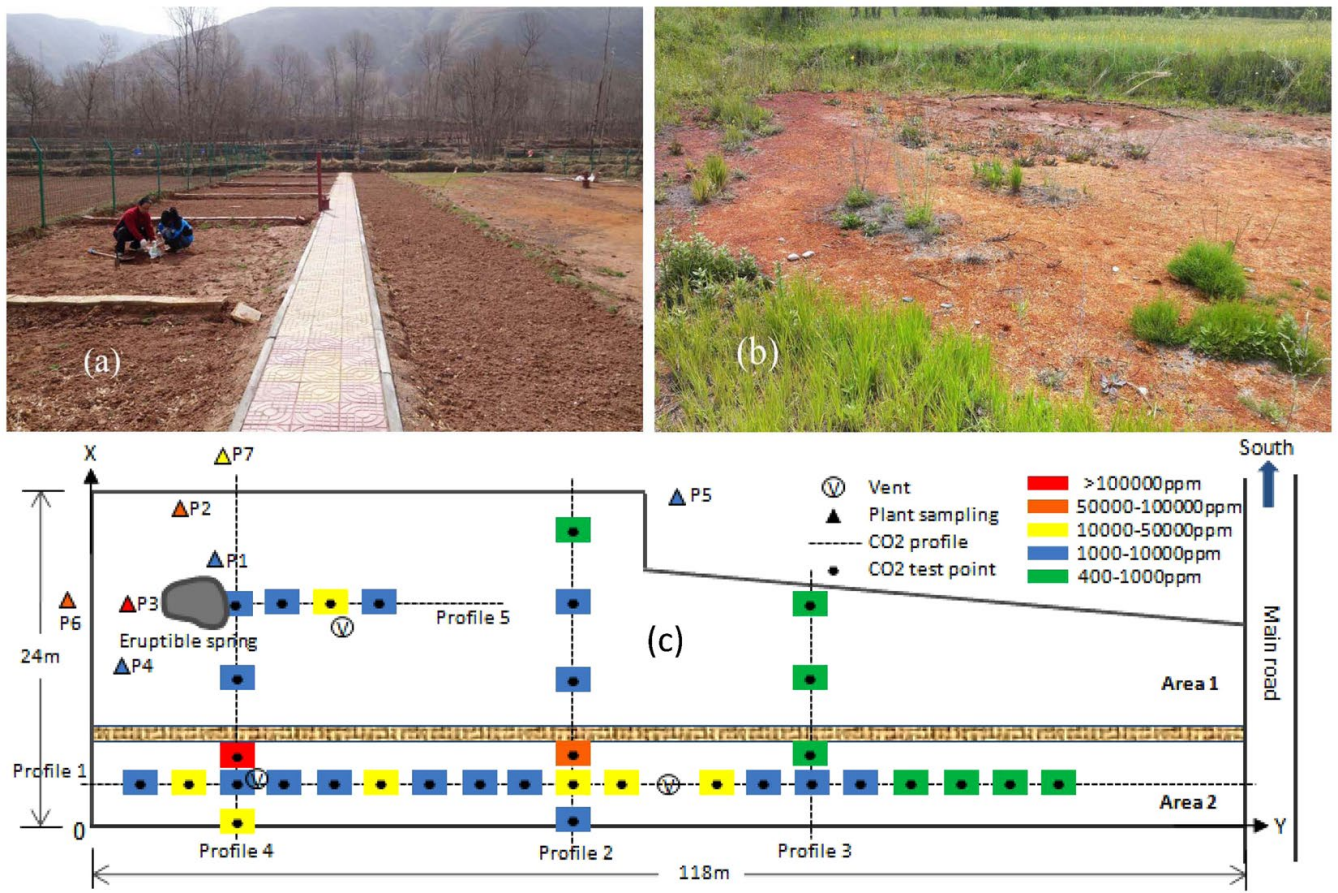
Photographs of the CO_2_ Research Field (**a**,**b**), and the illustration of plant investigation points & CO2 test strategy and concentration ranges (**c**).

Precipitation mainly falls in the growing season (June to August), which coincides with the highest temperatures. A minimum monthly mean air temperature is −18.7 °C in January and the maximum of 25.13 °C occurs in July. The site has a river gravel layer covered with 30–40 cm deep cultivated loess soil. The local crops mainly consist of highland barley, wheat, rapeseed and potato (Fig. 5a).

In the CO_2_ research field, there are four main gas vents. One is an intermittently eruptible spring (Fig. 5(b)) and the other three are abandoned water wells. Within the gas vents, there are high CO_2_ flux rates, as well as nearly 100% CO_2_ plus very small portion of trace gases such as CH_4_, H_2_S and H_2_. Due to acid water (pH = 5.2) flooding from the eruptible spring, a serious acidification of the surface soil in the middle of Area 1 occurred, resulting in a no-vegetated zone west of eruptible spring. Although the age of this gas vent is not exactly known, interview with local residents indicates that it has been active and stable for more than 10 years. Therefore, except some wild plants living in Area1, there are no local crops planted in this field.

### Sample collection and preparation

As shown in Fig. 5(c), the plant investigation points were marked as P1 to P7, and five transects were selected to define the spatial distribution of CO_2_ concentrations in the shallow soil (20–30 cm deep). CO_2_ concentrations were measured with portable infrared CO_2_ analyzer (GXH-3010 E1, Huayun Beijing). Then, based on the information of CO_2_ concentration profiles, a total of 6 points with different X and Y scales (as listed in Table 1) were selected for soil sampling, which were subsequently analyzed for various chemical and biological parameters. The two blank samples marked as S001 and S002 (Table 1) were taken from the farmland surrounding the field.

When sampling, all the wild plants and grass above the soil surface were removed and soil was extracted from 20–30 cm depths, as well as the plant root zone. After collection from the site, the soils were air-dried then gently crushed by hand and sieved through a 2000 um sieve to remove large roots and rock fragments, vegetable matter, and other particles larger than 2 mm in size. The prepared samples were stored in clean plastic zip-lock bags for the following tests and analysis.

In terms of sampling for biological tests, all tools and bags were cleaned with alcohol ‘disinfectant’ wipes. The samples were stored in a refrigerator and analyzed as soon as possible.

### Soil chemistry

Ten gram of air-dried soil were put into a 50 ml beaker, then 25 ml de-carbon dioxide water was added. After stirring 1–2 minutes and settling down for 30 minutes, the pH of supernate, as well as soil pH, was determined using a pH electrode.

According to standard procedures, total nitrogen (TN) was determined using an automatic determination instrument of Nitrogen (Foss Kjeltec 8400, Danmark), ammonia (NH_4_^+^), total phosphorus (TP) and readily available phosphate (AP) were analyzed by the Smartchem Discrete Auto Analyzer (ADA, CleverChem200, Germany). Soluble salt ions like Ca^2+^, Mg^2+^, Cl^−^, SO_4_^2−^, CO_3_^2−^, HCO_3_^−^ were determined by standard methods of titration^56^. X-ray diffraction (XRD) (D/MAX-3C Japan) was adopted to investigate the change of mineralogy.

### Botany

Since there are no plants in Area 2, botanical surveys were conducted in Area1. As shown in Fig. 2, four 1 × 1 m quadrats (named as P1, P2, P3 and P4) surrounding the eruptible spring were selected to investigate the distribution of main plant groups and measure the plant growth character indexes, as well as total weight, length of root, height of stem and number of leaves.

Because of existence in all plots, *Argentina anserina* and *Plantago asiatica* were chosen to explore stress tolerance under different CO_2_ concentrations. We picked some leaf samples and stored them in a refrigerator for the following test. Adversity resistance indexes such as catalase (CAT), peroxidase (POD), superoxide dismutase (SOD) and proline (PRO), photosynthesis identified by chlorophyll *a, b* and carotenoids concentrations were determined. Plant quality indicators like soluble protein and surge were measured as well.

The typical local crops are wheat, rapeseed and potato. Therefore, we collected samples of wheat berry, rapeseed and potato at plant collection points (P5-P7) surrounding the research filed (shown in Fig. 5(c)) and blank crops were taken from near farmlands, which are in the same valley with the research field, for soluble protein, sugar, fat and dietary fiber tests. All the tests described above had five parallel samples and were conducted according standard methods^57^.

### Microbiology

According to standard monitoring methods^58^, soil dwelling animals were investigated in blank soil and CO_2_ invaded soil. Each soil has 9 monitoring plots of 60 cm diameter and 30 cm depth. Based on the traditional plate count method and advanced molecular biological approach, the soil bacteria and fungi diversities in different concentrations of CO_2_ were investigated. By using Denaturing Gradient Gel Electrophoresis (DGGE), microbial community structures in soil samples were analyzed. The main procedures include microbial DNA extraction and purification by using extraction Kit Ver20 (TaKaRa), amplifying target DNA to get PCR product. Thereafter, 16 S rDNA-DGGE fingerprint spectrums of the soil samples were obtained via Gel Imaging System. Then, DGGE bands were recovered for PCR amplification, followed with TA clone of target fragment. Subsequently, fractional 16 S rDNA genome sequences of recovered bands were tested and bacterial community structures were identified.
